# Examining impact of service user involvement: a follow-up study of user-led mental health service evaluation

**DOI:** 10.3389/fpsyt.2025.1621918

**Published:** 2025-10-13

**Authors:** Hilda Näslund, Katarina Grim, Urban Markström

**Affiliations:** ^1^ Department of Social Work, Umea University, Umea, Sweden; ^2^ Department of Social and Psychological Studies, Karlstad University, Karlstad, Sweden

**Keywords:** mental health, service user involvement, user-focused monitoring, service evaluation, impact

## Abstract

User-focused monitoring (UFM) is a method for service evaluation led by service users, aimed at enhancing quality and service user involvement. Our study examines the experienced outcomes following the completion of five UFM projects within Swedish mental health service programs. Specifically, we focus on the barriers and facilitators to integrating user-led evaluations into service program development. Through interviews with user monitors, managers, staff, and service users involved in these evaluation projects, we conducted a directed content analysis focused on preconditions, processes, and outcomes. Our findings indicate that UFM projects contribute to specific development measures and improved relationships within service programs but also to broader cultural change. However, a lack of trust among service users in actual change poses a significant obstacle to their involvement. Additionally, the absence of feedback regarding the implementation of change measures by service programs to service user groups risks reinforcing this lack of trust. To enhance the integration of evaluation results into quality development, early engagement with staff groups and clear implementation plans are recommended.

## Introduction

Mental health services are in many countries evolving toward a person-centered care approach (1), emphasizing recovery orientation and empowerment (2, 3). This highlights the importance of service user (SU) involvement and integrating experiential knowledge in practice. Despite policy support for SU involvement in Sweden (4, 5) and internationally (6, 7), sustainable implementation remains a challenge. The aim of this study is to explore the outcomes of user-led evaluations in the development of service programs, guided by the following research questions:

- What outcomes do service users, staff and managers report as a result of user-led evaluations within service program development?
- What factors are perceived as facilitators and barriers to the integration of user-led evaluations into mental health practice?

Our case for examining outcomes of SU involvement methods is User-Focused Monitoring (UFM), a user-led service evaluation method employed in Sweden. Similar methods are utilized in Norway (8) and England (9). In Sweden, UFM traces back to the 1980s and 1990s when SU organizations participated in developing a model for evaluating social services from a SU perspective (10). Internationally, many examples of SU involvement in service development – similarly to these early initiatives – focus on engaging SUs in assessments and quality improvements (e.g. 11, 12), yet the ownership remains with service or research organizations. In Sweden during the early 2000s, several projects further fueled UFM adaptation. These projects enabled the SU movement to create their own UFM models, allowing autonomous execution by SUs. Now, there are UFM models where individuals with lived experience lead the evaluation process at every stage (13–15). However, research on user-steered methods like UFM remains limited.

### The Swedish context

The development of UFM in Sweden is linked to the rise of various SU involvement methods like peer support (16, 17), user advisory boards (18), and shared decision-making (19, 20). The Swedish SU movement has been pivotal in shaping and maintaining these methods, operating similarly to external consultants by providing peer support workers and conducting UFM evaluations for public sector actors. Sweden’s UFM context has been characterized as a social democratic welfare regime (21), with public sector actors playing a dominant role in mental health services despite the emergence of private and nonprofit providers. The system is marked by sectorization: municipal social services handle housing, employment, and social needs, while regional healthcare services focus on specialized care and psychotherapy. Approximately 30 UFM projects are conducted annually in Sweden’s mental health sector, for instance focusing on accommodation and vocational services in municipal social psychiatry, and both inpatient and outpatient services in regional psychiatry. Most UFM projects occur in metropolitan areas with a strong SU movement (22). Some SU organizations have secured annual agreements with local authorities for a set number of UFM projects, ensuring predictable funding and stable employment for user monitors (22). Conversely, organizations that must seek funding for each project face challenges in maintaining consistent operations.

### User-focused monitoring

UFM systematically and externally evaluates service programs, units, or interventions from a SU/patient perspective. Typically commissioned by service providers, often managers, UFM is conducted by individuals with lived experience of mental ill health or their relatives, acting as user monitors. These user monitors receive training in basic evaluation methods, such as interview or survey techniques. Their experiential knowledge and peer identity ensure equitable interactions with SU/patient participants and contribute valuable insights throughout the UFM process, from question design to result analysis. Additionally, the role of a user monitor provides competency-building opportunities and work-life experience. In Sweden, various UFM models are practiced, some primarily based on interviews and others on surveys (13, 14, 23). Multiple actors, including SU organizations, R&D units, work integration social enterprises, and public sector actors, conduct UFM. To emphasize their higher degree of user autonomy, the SU movement describe their models as ‘user-steered UFM’ (13, 14, 23). However, prior studies have revealed variations in actual user autonomy across UFM projects (24). Typically, a UFM coordinator collaborates with the commissioning manager to plan the project. User monitors then gather data, typically involving around ten SU/patient participants, though this number can vary significantly based on the type of service being evaluated and according to UFM model (22). Staff play a key role in recruiting participants (24). Evaluations cover themes like personal treatment, participation, accessibility, competency, collaboration, continuity, activities, facilities, and food, focusing on SU perceptions and potential improvements (22).

This study addresses the call for research on the impact of involvement methods on organizational development (6, 25–27). Previous studies have analyzed the impact of SU involvement across various dimensions. Sandvin Olsson et al. (25) emphasize understanding the broad impact of involvement initiatives, including both the process and resulting organizational changes. Similarly, Gathen et al. (26) discuss the impacts on stakeholders and organizational changes. This study uses Gradinger et al’s (28) value system to evaluate the outcomes of user-steered evaluations across three dimensions: substantive outcomes, process outcomes, and normative outcomes. Substantive outcomes track concrete changes in service programs based on UFM input. Process outcomes examine the effects on participants and changes in service provider-recipient relationships. Normative outcomes explore broader cultural changes within services (28). While many studies emphasize the need to study objective effects or change measures (6, 27, 29), Gathen et al. (26) drawing on Banks et al. (30), propose viewing the impact of patient participation as circular rather than linear to capture its nuanced effects. Moreover, a critical concept in SU involvement research is tokenism (e.g. 31, 32), introduced by Arnstein (33), which analyzes symbolic rather than substantive involvement where decision-making power is not transferred (34). Tokenism is key in analyzing changes induced by UFM projects.

## Materials and methods

The study was conducted in collaboration with a reference group with representatives from the SU movement and mental health service organizations, bringing experience from both provider and commissioner perspectives of UFM. The reference group met digitally approximately four times per year throughout the three-year duration of the project and was actively involved in all phases of the research process. Their engagement spanned from the formulation of research questions and the selection of case studies to the development of strategies for communicating the findings. The group reviewed and provided input on all publications produced within the research project.

### Data collection procedures

The study is part of a project focused on UFM. Previously, we followed the implementation of five UFM projects; this study continues by following up on their results. Reference group input, further informed by an earlier UFM mapping study in Sweden (22), played a key role in sampling decisions. We aimed for diversity across UFM projects, including evaluations of region-, municipality-based, and non-profit service programs. We sampled both commonly associated UFM contexts (e.g., accommodation services) and less frequently evaluated ones (e.g., forensic psychiatry). Interview methods are predominant among UFM projects within the mental health sector (22). Four projects used interview methods exclusively, while one used a mixed-method approach. Most UFM projects are conducted by large SU organizations with extensive UFM experience; we included three such projects. To broaden perspectives, we also sampled a project led by a work integration social enterprise and one based on a collaboration between a smaller local SU organization and a national SU organization. **Table 1** provides details on the service programs, UFM methods, and conducting organizations.

**Table 1:** Overview of the included UFM projects.

Service setting	UFM method	UFM provider
UFM 1. Housing support in large city	Interviews with 15 SU participants	Work integration social enterprise
UFM 2. Housing with special services in metropolitan area	Interviews with six SU participants	Large local SU organization
UFM 3. Non-profit user-run daily activities program in a metropolitan area	Interviews with 12 SU participants	Large local SU organization
UFM 4. Forensic psychiatry hospital in a small town	Mixed method, 58 survey responses, 14 in-depth interviews	National SU organization in cooperation with local SU organization
UFM 5. Child and adolescent acute psychiatric unit in a metropolitan area	Interviews with six SU participants	Large local SU organization

We conducted interviews with key actors across the five UFM projects at three stages: 16 interviews with user monitors/UFM coordinators and managers during the start-up phase, five group interviews with user monitors/UFM coordinators upon project completion, and 25 interviews with managers, staff, and SUs/patients 6–12 months post-completion. **Table 2** provides an overview of the data collection process at these stages.

**Table 2:** Overview of the sources used in each data collection phase at each of the service sites in this study.

The UFM-cases	1. Start-up data collection phase	Interviews with user monitors	2. After completion data collection phase	UFM report	3. Follow-up data collection phase	Interviews with staff	Interviews with SUs/patients
	Interviews with managers		Group interviews with user monitors		Interviews with managers		
UFM 1	2 unit managers	2 user monitors	3 user monitors	1 UFM report	1 unit manager	2 staff members	3 SUs
UFM 2	1 unit manager	2 UFM coordinators	2 UFM coordinators	1 UFM report	1 unit manager	2 staff members	3 SUs
UFM 3	2 board members	1 UFM coordinator	5 user monitors 1 UFM coordinator	1 UFM report	1 board member	2 activity leaders	2 members
UFM 4	1 section head	2 user monitors	1 UFM coordinator 3 user monitors	1 UFM report	1 section head	2 staff members	2 patients
UFM 5	1 acting unit manager	2 user monitors 1 UFM coordinator	2 user monitors 1 UFM coordinator	1 UFM report	2 unit managers	1 staff member	1 user monitor Complementary questions to staff member

In previous studies, we explored the start-up phase (24) and the implementation process (35) of UFM using data from the first two phases. This study shifts focus to the follow-up phase of these five UFM projects, examining barriers and facilitators to integrating user-led evaluations into service program development. To understand the entire UFM process, we integrate data from the first two phases as background, but emphasize the third phase to explore UFM outcomes from the perspectives of SUs/patients, staff, and managers.

For detailed descriptions of data collection during phases one and two, see Näslund et al. (24) and Näslund et al. (35). During the follow-up phase, we interviewed managers, staff, and SUs/patients involved in the projects. Managers, previously interviewed during the start-up phase, informed staff about the possibility of participating in the study. For recruiting SUs/patients, we employed various strategies. In some projects, user monitors shared study information with those who participated in evaluations, in others service providers also posted information on social media. In UFM 5, targeting a child and adolescent acute psychiatric unit, user monitors informed patients, but none were interested in participating, perhaps due to the young age of the patient group and the acute nature of the service program. To include patient perspectives, we interviewed a user monitor and asked patient-focused questions during staff interviews. During the semi-structured interviews, we focused on the actors’ experiences with the UFM project, how results were communicated to the service provider/SU group, how UFM findings were integrated into service quality development, and specific changes resulting from the evaluations. We provided both written and verbal information about the study to all interviewees, obtained written informed consent, and received approval from the Swedish Ethical Review Authority (ref. 2021-02550).

### Analytical procedures

In this study, directed content analysis was chosen as it offers a systematic and transparent approach to identifying patterns, themes, and meanings within qualitative interview data. This approach is particularly suitable when prior research or concepts can guide initial coding categories, while still enabling the identification of emergent themes that extend or refine current understanding (36). Each UFM project was analyzed as an individual case, focusing on preconditions, processes, and outcomes of the evaluations as predetermined categories (36). Data from the first two phases were integrated, especially for assessing preconditions and processes, but our primary emphasis was on the third phase to explore UFM evaluation outcomes. The first author led the analysis, with active participation from all authors and input from the reference group. Below, we present the results structured by the above mentioned phases, and the five distinct UFM projects.

## Results

### UFM 1: housing support

#### Preconditions and process

UFM 1 evaluated a housing support program in a large city (**Table 1**). Conducted by a work integration social enterprise, the assessment spanned six months and involved interviews with 15 SUs to gather insights on their collaboration with housing support workers. This focus was set to evaluate ongoing development work in this area. The manager emphasized the necessity of political support to ensure the adoption and sustainability of the method. In this region, obtaining evaluation funding is currently difficult, underscoring the importance of political involvement for securing long-term commitment to UFM.

UFM 1 showcased a process that, to a higher degree, was steered by the service provider. Managers set the focus of the UFM and omitted certain interview questions during initial meetings with user monitors. Despite this, user monitors appreciated the project’s clear scope. Staff were not involved in commissioning or planning but played a crucial role in recruitment. SUs received support within their homes, posing challenges for collective communication about the UFM project. Staff engagement was thus crucial for successful recruitment. Some staff members were, however, unaware of the ongoing UFM project, indicating a lack of vertical integration. User informants had positive experiences with the UFM, feeling safe and valued by the user monitors. The user monitors emphasized that their lived experiences were vital in building trust and reducing power imbalances. However, some user informants were unaware of the user monitors’ backgrounds. This was the involved user monitors’ first UFM project, and they noted that the analysis was time-consuming and that they could not bill for all the hours spent on the project.

#### Outcome

The user monitors presented the UFM report at workplace meetings. The manager saw the results as confirmation of their current development work:

It felt like we are indeed heading in the right direction with this ongoing change journey. So, we received considerable credit, affirming that, yes, we are on the right track. In that sense, we continue along the same path. Manager

The results were further discussed as a morale boost for staff, who appreciated the positive feedback. However, staff noted that the small number of participants limited the generalizability of the results.

The manager discussed how they had addressed improvement suggestions by strengthening the focus on SUs’ social needs during care planning and implementing systems to target specific life areas. New routines were introduced to streamline routines for substitutes and to address SUs’ lack of knowledge about where to direct complaints or suggestions. Despite these efforts, the manager acknowledged the difficulty in attributing specific improvements solely to the UFM, a view shared by staff: *“It’s not like because of … the improvement proposal here, really. It’s not that. But it has come anyway”* [Staff 1]. However, the process reinforced their commitment to ongoing development work. The manager suggested the need for proactive planning for better utilizing the results: *‘I believe we should have devised a plan … How would we proceed upon receiving the report? What steps should follow?’* [Manager]. Staff members expressed that them being more involved in analyzing the results and how to address these would have been valuable. Staff further found implementing UFM alongside ongoing development work challenging but recognized broader benefits, such as ensuring SU voices are heard. However, they were uncertain whether the SU group received the results or any information on their implementation:

For instance, they might wonder, “What has been done with this?” It’s a relevant question. Somewhere, a service user participating in these processes might also desire some form of follow-up – to know if their input led to any tangible outcomes. Staff 1

User informants did not receive the report or any reporting of the results. They further described not being informed about any changes made by the service provider in response to the UFM but stressed the importance of acting on feedback:

If there are things that need to be changed or updated, adjusting routines based on what emerges from this – well, I think it’s crucial. Otherwise, it’s all in vain to have this UFM. Then it’s like you do the UFM to be able to say that we’ve had a UFM. Then you discard it, only to repeat the same process some years later. So, I genuinely hope they take the feedback to heart. Otherwise, it’s a waste of time for everyone involved. And I imagine that some of those participating truly want … People who’ve had negative experiences want to see real, noticeable change. At the very least, some form of feedback.’ SU 1

User informants described no significant changes following the evaluation, but many were satisfied with the existing services. They valued having their voices heard and noted that UFM fosters mutual understanding in the care recipient-provider relationship. All groups agreed on the value of continuous UFM projects. Although a follow-up visit by user monitors was planned, it did not occur, possibly due to insufficient funds.

### UFM 2: housing with special services

#### Preconditions and process

UFM 2 was conducted within an accommodation service program by a large SU organization with extensive UFM experience. This organization has an agreement to conduct several UFM projects annually within municipal services. The accessibility of SUs and staff streamlined this UFM process. User monitors informed both staff and SUs about the upcoming UFM during workplace and house meetings. The project was based on interviews with six out of eleven residents and spanned six months. The manager described valuing the user monitors’ experiential knowledge and autonomy, allowing them to lead in formulating questions. Staff also emphasized the UFM as the SU’s platform, to express their views. Expectations of change varied among user informants. Some believed the existing program was already good, while others desired improvements but were skeptical about the feasibility of actual change.

The manager noted that the service program previously lacked staff continuity. Recently, the team had become more stable and cohesive, making them ready for an evaluation. Conducting a UFM requires a mature staff group capable of focusing on broader objectives. Sound anchorage was discussed as key to effective utilization:

ensuring that everyone is on board so that we can work with it in the best possible way. So … Yes, I believe that might be the key as well – not just having one person engaged or relying solely on the manager to drive it. It’s essential that this isn’t something done in isolation but rather utilized effectively. Manager

Staff also emphasized the importance of understanding the UFM method and its objectives. The user monitors’ collaborative approach, focused on quality improvement, was highlighted:

They also mentioned this during the introductory meeting with the staff. The idea is that we work to ensure that the SUs have a voice and can express their perspective. The whole purpose is not to point out faults but to ensure quality and make sure we’re focusing on the right things. Manager

The project used a drop-in approach, allowing SUs to participate directly at the service site, complementing pre-booked interviews. Staff played an important informational role, encouraging SU involvement. SUs described the UFM as a positive experience, valuing the user monitors’ lived experience, which contributed to their comfort during interviews.

#### Outcome

The UFM findings were shared through presentations to the SU group, staff, and the manager, and a poster at the service site. Overall, the results were positive, and the manager saw them as validation of the staff’s work. Staff appreciated the balanced critique from user monitors, which facilitated constructive learning:

They also present what is less favorable in a very professional manner, allowing one to absorb it within the professional context. It doesn’t become as charged as when someone might stand and shout their disappointment. It remains very professional. Staff

The approach ensured the results were well-received rather than confrontational. However, staff noted the presentation emphasized positive outcomes despite some alarming findings, highlighting the need for stricter feedback from user monitors.

Based on the UFM results, it was observed that certain SUs expressed concerns about safety due to previous violent incidents. This prompted internal discussions and led to the development of an action plan. Activities were identified as an area for improvement, leading to measures such as the installation of pallet collars, the organization of summer activities, and the implementation of biannual excursions. The appointment of substitutes was also identified as a problem area. Efforts were made to enhance staff stability and training. Ensuring staff delivered on promises to SUs was a primary focus, continually discussed during workplace meetings and with new procedures established for translating information, ensuring that SUs received timely feedback. A significant challenge was the lack of work and employment opportunities for the SU group. Efforts were made to inform them about available options. Broader outcomes also emerged, as both staff and manager underscored the importance of gaining deeper insights into the SU group. This allowed them to better comprehend the reasons behind SU critiques and expressions of dissatisfaction, ultimately improving relationships. Staff members also reported that the UFM had challenged their perceptions of the SU group, expressing surprise at the high level of participation interest shown by the group.

SUs reported not receiving explicit feedback on actions taken based on UFM results, and reported varied experiences with changes, noting improved communication and feedback from staff but inconsistent follow-through with regard to activities. Looking ahead, the manager as well as staff, emphasized the importance of effectively communicating action plans for UFM results to the SU group:

they should receive confirmation that their feedback has been addressed, or that we have taken care of the issues they informed the user monitors about. However, I think we haven’t been as effective as we would have liked. Even though we’ve worked within the processes, I wonder: How much have we actually provided feedback? Manager

Six months later, user monitors reconvened with the manager for a follow-up meeting, which was valuable for refocusing on results. Staff would have appreciated participating in the follow-up, the manager also stressed the need for involving staff in developing action plans. All groups emphasized the need for consistency in UFM projects:

I would have done it several times because I think it was very rewarding. But it was rewarding for a while. It brought a lot of things to the back of my mind. Thoughts and areas for improvement and so on. But concretely, follow-up is needed for it to really be carried forward. Staff 1

### UFM 3: non-profit user-run daily activities program

#### Preconditions and process

UFM 3 was conducted within a non-profit, user-led daily activities program (**Table 1**) by a large SU organization spanning around six months. The SU organization had stable funding, through an overarching agreement. The program’s activities, focused on physical activities and sports, were spread across the city, making it challenging to inform all members about the UFM. Out of 150 members, 12 participated in interviews. The UFM aimed to enhance activities by providing the association’s board and staff with deeper insights into members’ attitudes and experiences. The chairman further saw the UFM as a way to increase member influence, create communicable material for external stakeholders, and set tangible improvement targets. Staff anticipated honest feedback.

A common challenge in UFM, recruiting SUs for the evaluation was the most challenging part also for this project. Activity leaders informed members about the UFM, and social media channels were used to disseminate information. Staff emphasized the importance of providing clear information about the UFM’s purpose and process, highlighting its value for participants. Both service program representatives and members reported a positive experience with the UFM process. They appreciated the active involvement of user monitors. The chairman emphasized that the UFM method enhances evaluation validity due to its professional and independent approach.

#### Outcome

The UFM results were presented to the board and activity leaders, and the report was distributed to members via email, social media, and at the annual member meeting. The chairman acknowledged the difficulty in assessing the reach due to the program’s decentralized nature. The overwhelmingly positive results boosted staff morale:

We felt uplifted and happy about this. Still, we can bring it up at certain meetings, saying, ‘Remember the great feedback we received, and what we do is very important and highly appreciated by the participants and so on.’ Staff 1

Being able to report positive outcomes to external funders was also seen as a significant gain:

it was good that we thereby could demonstrate positive results to them because they are one of our principals. So, in that sense, it’s actually the most concrete result we’ve obtained. We’ve proven to them that we’re doing a good job and that it’s beneficial for them to invest further, allocate financial resources to support us. Chairman

The chairman was surprised by the positive feedback, having anticipated more criticism. However, the results highlighted areas for improvement, particularly in the variety of activities and the ability to influence them. To address this, the program hired a new activity leader to increase scheduling flexibility and planned to engage members about their preferred activities through surveys. Staff addressed issues like excessive competitiveness and inconsistent instructions from activity leaders through collaborative discussions. Despite these efforts, the chairman noted that focus on the results eventually waned:

We received very positive feedback, so everyone was very happy about it. Later, we brought it up during a board meeting, and discussed it there. Since then, we haven’t really talked about it much. Chairman

Members noted a lack of explicit feedback on changes implemented following the UFM but some had been informed of modifications aimed at increasing participation. One user informant suggested that the relationship between activity leaders and members may have improved due to the UFM process, fostering greater equality. User monitors conducted a follow-up meeting exclusively with the chairman. Both staff and members recognize the importance of initiating new UFM projects, especially given the evolving activities and member base of the program.

### UFM 4: forensic psychiatry hospital

#### Preconditions and process

UFM 4 evaluated a large forensic psychiatric hospital (**Table 1**). It involved collaboration between national and local SU organizations and utilized mixed methods, including interviews and surveys. The evaluation drew on 58 survey responses and 14 in-depth interviews (122 patients at the hospital in total). Spanning approximately one year, the primary goal was to explore care experiences, particularly for long-term patients. This project stood out for its high level of user autonomy. Service providers were involved initially but did not review interview or survey questions. Instead, user monitors collaborated with a council of patient representatives to prioritize the patient perspective.

Managers and staff hoped the UFM would empower patients and identify improvement areas. User monitors in addition to this, linked the UFM to social movement objectives, developing knowledge for evidence-based advocacy. Patients participated to share their experiences of forensic psychiatry, though their expectations for change varied. Some hoped for improvements, while others were skeptical.

Promoting the UFM across different care units required significant effort. Safety protocols restricted user monitors’ access, leading to digital presentations and informational flyers instead of direct patient contact. The lack of direct engagement with staff or the patient group led to implementation challenges. Information flowed from management to unit coordinators, then to staff, who informed patients. A critical issue was the lack of clear understanding among staff for the UFM project and its objectives: *“No, it wasn’t clear to everyone what this entailed. I didn’t get that sense”*, noted one staff member. Management recognized this as a crucial issue, particularly challenging in a large organization:

It’s difficult when it’s such a large workplace, and everyone is caught up in their own daily routines. When you work directly in the units, you don’t always have the full perspective on why we’re doing these things and their importance. What’s the purpose? … Yet it’s challenging to effectively convey the message and get people truly engaged in this type of project. Often, people might perceive it as an additional burden on top of their existing workload. Manager

Emphasizing the value of staff contributions and adapting the UFM to varying needs in different service contexts were also essential. Staff engagement varied, with some highly engaged and others less interested. Motivational efforts were necessary to convey to patients that their voices mattered:

There are individuals who are quite negative, and they have a rather pessimistic attitude that it doesn’t matter what I say; nothing will change anyway. But it’s about continually working with it and getting into this … this little bubble that has been created, where it actually makes a difference. It is possible to make a difference. What you say matters for your care. Staff 1

Patients cited distrust in authorities and the healthcare system as significant barriers to participation but reported positive experiences from UFM participation. However, managers and user monitors acknowledged conflicts and communication challenges in their collaboration. User monitors found that power relations hindered implementation, requiring a step-by-step approach. Interviews indicated that mutual trust between these actors had not fully developed. Managers described mixed experiences, including misunderstandings and a critical, suspicious approach from user monitors:

some form of mistrust and … It got a bit tricky sometimes when we had certain gatherings so … Then I felt that there was like … it wasn’t really expressed, but still that we as management in some way didn’t do what we should … We hindered patients and staff and yes but a little such insinuations which felt very tricky when we now chose to do this UFM and open up the service program and really gave room for it too. Manager

Mutual trust between stakeholders was stressed as a foundation for successful UFM implementation.

#### Outcome

The user monitors used various methods to report results back to the hospital, including short films and multilingual folders for patients, and a presentation for managers, to complement the UFM report. Managers described how they had preferred more detailed yet accessible information for patients. Some formulations in the report were further discussed by management as methodologically unclear, leading to tensions between user monitors and management. User monitors felt their experiential knowledge, methods, and results were questioned, while managers believed user monitors lacked a full understanding of forensic psychiatry. The manager acknowledged the need for more preparatory work. User monitors described how a form of power struggle ensued after reporting, with service providers asserting control over dissemination.

Key UFM findings included half of the patients expressing dissatisfaction with the food, prompting ongoing efforts in this area. Some patients found the care team system confusing and wanted a designated contact person, leading to a new system integrating these models. Staff worked on improving communication with patients. The most significant development was re-establishing a patient council:

We had a patient council that was inactive and not really functioning, so we took charge and started working on it with the help of [SU association] afterward. That’s what I can truly see as a benefit of doing this. Manager

A later survey showed higher patient participation. Staff suggested that the UFM might have contributed to this improvement. However, staff and managers found it hard to pinpoint specific improvements as many suggestions were rather integrated into ongoing work. Some staff did not recall the results and stressed the need to ensure outcomes are well received. They also highlighted the importance of maintaining focus on UFM results by planning for implementing changes. Nevertheless, they acknowledged the value of external evaluations, especially in the closed-off environment of forensic psychiatry. Although patients were not informed about the application of UFM results, some noticed positive changes and valued learning about SU organizations. Other patients saw no significant changes and cited persistent hierarchies as obstacles:

I don’t really see much of a difference … As I said, it’s a very closed world, and it’s difficult to see any significant changes after a UFM, unfortunately. And it’s indeed a very solid pyramid in a hierarchy. It is [the clinic manager] at the top, and we patients are at the bottom. Patient 1.

Patients emphasized the importance of continuous UFM to observe tangible progress. User monitors returned for a follow-up meeting with management.

### UFM 5: child and adolescent acute psychiatric unit

#### Preconditions and process

UFM 5 was implemented in a child and adolescent acute unit in a metropolitan area (**Table 1**). Care duration varied, with some receiving long-term care and others shorter stays. Conducted by an experienced SU organization under an overarching agreement with local authorities to conduct UFM, the UFM aimed to assess the service program from the SU’s perspective, identifying strengths, areas for improvement, and future development directions. Six young patients were interviewed and the project spanned just over one year.

The UFM coordinator visited a staff meeting to discuss the upcoming project, but only two staff members could attend due to the unit’s acute nature. The service program was undergoing significant changes, including management shifts. The initial manager received positive feedback from staff after this initial meeting but went on parental leave shortly after. An interim manager was appointed during the UFM implementation, followed by a third manager after its completion. Several factors contributed to difficulties during the project, including management changes, unit renovations and the organization’s high-pressure environment, with many staff members experiencing stress and several being on sick leave. This upheaval contributed to a lack of understanding among management and staff about the UFM method, as revealed in interviews.

Staff played a crucial role in motivating children and adolescents to participate. Despite continuous communication between the UFM coordinator and the manager, recruitment challenges arose, possibly due to understaffing, the contact person’s incomplete understanding of the mission, and the service’s focus on children and adolescents in acute situations. A user monitor further noted skepticism about real change among young patients as a deterrent:

Sometimes, when you approach people to recruit them for participation … you hear responses like, ‘Well, it won’t make any difference anyway.’ So, in some way, you need to emphasize that it is indeed possible to influence one’s healthcare, but then it must also be based on truth. User monitor

During interviews, the user monitor found the questions might have been overwhelming for the young participants and suggested the need to tailor interviews to this target group.

#### Outcome

During a workplace meeting, the UFM coordinator and a user monitor presented the evaluation results: They emphasized the importance of delivering balanced feedback to strengthen engagement and foster a sense of ownership and motivation for change. Despite some negative results, the presentation was constructive and non-judgmental, which the manager appreciated:

they were not accusatory but more understanding, and how we are going to be able to continue working with certain things. And it was positive, that it was not judgmental … Manager 2

Staff found the results presentation positive but struggled to recall specific details:

I only remember that we talked about it. Specifically, it’s like when you sat there and heard it, and it was just like, ‘Yes, but we can recognize ourselves in that.’ … I think it was something related to food and those kinds of things, where some people maybe thought it was a bit worse and so on. So, yeah, but I can’t recall exactly. Staff

This lack of recall meant they were unaware of measures taken to address the findings. One key focus area was care plans, highlighting the need to enhance understanding and active participation. The manager noted this as a motivator for staff:

In our UFM, patients have reported the following regarding care plans: they either don’t know what a care plan is or have one but don’t feel involved. So, this has also been a great motivator to encourage staff engagement and focus on improving care plans. Manager 2

The UFM results highlighted a lack of patient participation and recommended reintroducing Patient forums, an SU involvement method that had been paused. In response, the manager decided to restart the Patient forums. However, as with other UFM projects, the specific actions taken by service providers were not communicated to the young patient group.

## Discussion

Our findings suggest that UFM projects not only support targeted development initiatives and strengthen relationships within service programs, but also contribute to broader cultural transformation. However, a prevailing lack of trust among service users regarding the realization of change presents a substantial barrier to their active participation. Furthermore, the absence of feedback from service programs to service user groups concerning the implementation of change efforts risks deepening this mistrust. Key elements of the UFM method include trust and commitment from all stakeholders, protecting user autonomy, and independent external evaluations to enhance trust and legitimacy (13–15). Ongoing engagement with UFM is necessary to monitor the integration of insights into service enhancement efforts (13–15). These aspects align with this study’s findings on essential factors for successful implementation and optimization of UFM outcomes.

### Organizational challenges

Key results related to preconditions have been identified. Organizations under strain or undergoing change faced greater difficulties in implementing UFM. Stable staff and management were key facilitators for smooth implementation. A stable work group was crucial for an open and receptive approach to constructive criticism, where the team felt safe and mature enough to accept input through UFM. Hierarchies and unequal power relations hindered the implementation process. The findings suggest that organizations that had already integrated a co-production approach found it easier to implement UFM, aligning better with their existing practices. Similar patterns have also been raised in prior studies (37). Additionally, our results suggest that UFM projects were easier to conduct in service programs with spaces for the SU group to meet collectively, facilitating both start-up information and feedback. Delays in UFM results, often due to instability in involved organizations, were problematic as feedback was postponed. Financial resources were further central to conducting UFM. Previous studies have highlighted the importance of overarching agreements with public sector actors for executing UFM projects (22). The need for sufficient funding for sustainability in SU involvement in service development has also previously been raised (25).

### An oppositional approach and trust

Our study highlights different strategies for conducting user-led evaluations as the UFM projects varied in their focus on user autonomy. Gathen et al. (38) discuss strategies in SU involvement, ranging from oppositional to negotiating and cooperative approaches. Organizations conducting UFM often shifted between these strategies, with collaboration and negotiation dominating. A collaborative approach emphasized cooperation to develop services and enhance SU involvement, while a negotiating approach balanced critical and collaborative strategies to maintain relationships with public sector actors. In one project, an oppositional approach was observed, where user autonomy was more pronounced, and service providers felt user monitors were critical of their involvement. These strategies come with various losses and gains. Negotiating or collaborative approaches build relationships between commissioning and providing organizations, fostering a positive attitude toward utilizing results and conducting additional UFM projects. However, UFM relies on honest critical appraisal, and too much dependence on positive relationships can compromise autonomy and co-opt SU perspectives. Conversely, oppositional approaches allow for higher degrees of critical appraisal but carried the risk of user monitors being perceived as ‘unprofessional’ by service providers. Our results suggest that failing to establish collaborative relationships makes it less likely for results to be welcomed by service providers and reduces the likelihood of future UFM projects. Prior studies have highlighted how frames for what is defined as ‘professional’ can limit the knowledge and perspectives generated from SU involvement (39, 40). Anchoring in the SU movement and employing a clearly defined method with established parameters for input from service providers is crucial for maintaining sufficient autonomy for SU organizations conducting user-led evaluations in collaboration with service providers.

### Anchorage and planning result implementation

A previous study highlighted the importance of ensuring sufficient anchorage among all involved actors, particularly staff and the SU group, regarding the multiple purposes of conducting UFM (24). This finding is also key in the current study. Although staff and the SU group are often only minimally involved in the early planning phases of a UFM project, they are crucial for its implementation. The UFM method, connected to numerous purposes, requires anchorage among all actors. To achieve this, both vertical and horizontal integration of methodological procedures and purposes is needed (cf. 41). Several staff members reported a lack of anchoring, leading to difficulties in distinguishing UFM from other SU involvement methods and recalling concrete results. Investing time in thorough discussions on the value and procedures of UFM during the start-up phase is crucial.

A key finding is that UFM evaluations risk underutilization if service programs lack structured plans and procedures for applying the results, potentially leading to tokenistic involvement. Most service programs valued UFM feedback and described change measures. Participating SUs in several UFM projects reported improved relationships with staff, due to changes in communication methods or feeling their feedback was valued. UFM projects also positively impacted staff motivation and engagement, boosting morale when positive results were received. These outcomes can be seen as *process outcomes* for stakeholders (cf. 25, 26, 28). Following UFM evaluations, several concrete improvements were implemented, such as enhanced complaint handling, better information and feedback systems, hiring new staff, reorganizing existing staff, and establishing or revitalizing forums for SU influence. These organizational changes could be discussed as *substantive outcomes* (28). However, attributing these changes directly to UFM was challenging, as they often aligned with broader ongoing development efforts. This pattern relates to the risk of service providers mainly engaging with improvement suggestions already identified in their internal development work. Several interviewees described risks of UFM results being lost. Establishing a clear implementation plan with follow-up procedures could ensure better utilization of UFM.

### Feedback and building trust in actual change

A key finding was that none of the service programs provided feedback to the SUs on how they planned to use or had used the UFM results. Some SUs observed positive changes post-evaluation, while others did not notice improvements. Some were content with existing services and saw no need for changes. However, all SUs expressed a desire for feedback on how the UFM results were utilized. Several noted that the lack of information could decrease their motivation to participate in future initiatives. This feedback is crucial, as most interviewees, despite participating in the UFM, expressed a lack of trust that their involvement would lead to any change. This pattern is related to ‘consultation fatigue’ (42), where a lack of feedback on how input has led to changes can make participation feel meaningless and reduce engagement. Trust among all actors is a key dimension in UFM and the importance of service providers’ trust in the method has been highlighted (24). These results emphasize the necessity of trust between the SU group and service providers. The SU group need positive experiences from participating in UFM (process outcomes) and seeing evaluations lead to actual changes (substantive outcomes) to build such trust. This can contribute to empowerment processes, where SUs recognize their crucial role in service development built on co-production.

UFM evaluations lay the foundation for organizational learning. Integrating SU groups into these processes can be seen as radical or transformative learning (43), involving changes in perspectives, working methods, and challenging societal power structures. For UFM to achieve broader *normative outcomes* (cf. 26, 28) continuity and gradual re-negotiation of roles and culture are needed. Similar to Gathen et al. (26), we argue that creating impact through UFM should be seen as a circular, rather than linear, process. UFM can contribute to process outcomes for stakeholders and substantive organizational changes. Ensuring these positive impacts can support broader social change through gradual normative impact.

Finally, we wish to draw attention to the challenges associated with applying UFM within services aimed at children and adolescents. Such services often operate over shorter timeframes, which complicates the provision of feedback to young clients regarding the long-term outcomes of evaluations. Young individuals may also lack confidence in the value of their voices and opinions, a perception that is reinforced when they are not shown the impact of their responses. In our case study, some of the evaluation questions were at times perceived as overwhelming. We therefore identify a need to recruit and train young user monitors who are better positioned to engage with young service users, and to ensure that UFM approaches and questions directed at youth-oriented services are carefully tailored to the target group.

## Conclusion

Our findings indicate that UFM projects have three main types of impact. First, user-led evaluations can enhance relationships within service programs and empower participants. Second, they can lead to tangible improvements based on SU input. Third, they can foster broader culture change and support developments toward quality development built on co-production. However, a significant obstacle is the lack of trust among SUs in actual change, reinforced by the absence of feedback on the implementation of changes. To better integrate evaluation results into quality development, early engagement with staff and clear implementation plans are recommended. Continuity in UFM work is crucial for establishing a clear understanding of its purpose among all stakeholders, ensuring regular follow-ups, and reactivating results. Observing continuous impact is also essential for building trust that UFM will lead to actual change. Our results indicate difficulties in implementing UFM evaluations in contexts characterized by high staff turnover and a lack of continuity within services, as well as challenges in adapting the methodology to services targeting young individuals. The results also indicate that a shortcoming of UFM evaluations is their time-consuming nature. Additionally, the findings suggest that the most substantial impact is achieved in service programs that already exhibit ‘high quality’ in terms of a well-functioning work group and a strong interest in enhancing participation. Based on these findings, it may be advisable to primarily apply UFM to the refinement of relatively well-functioning services. Its structure and approach appear to be more conducive to enhancing existing practices than to initiating development in services facing more substantial challenges.

## Data Availability

The datasets presented in this article are not readily available because the dataset contains sensitive personal data, only anonymized data can be made available. Requests to access the datasets should be directed to hilda.naslund@umu.se.
